# Cyanogenesis in *Macadamia* and Direct Analysis of Hydrogen Cyanide in *Macadamia* Flowers, Leaves, Husks, and Nuts Using Selected Ion Flow Tube–Mass Spectrometry

**DOI:** 10.3390/foods9020174

**Published:** 2020-02-11

**Authors:** Hardy Z. Castada, Jinyi Liu, Sheryl Ann Barringer, Xuesong Huang

**Affiliations:** 1 Department of Food Science and Technology, The Ohio State University, 2015 Fyffe Road, Columbus, OH 43210, USA; barringer.11@osu.edu; 2 Department of Food Science and Engineering, Jinan University, Guangzhou 510632, China; ljy@stu2016.jnu.edu.cn

**Keywords:** *Macadamia*, hydrogen cyanide, cyanogenic glycosides, headspace analysis, SIFT-MS

## Abstract

*Macadamia* has increasing commercial importance in the food, cosmetics, and pharmaceutical industries. However, the toxic compound hydrogen cyanide (HCN) released from the hydrolysis of cyanogenic compounds in *Macadamia* causes a safety risk. In this study, optimum conditions for the maximum release of HCN from *Macadamia* were evaluated. Direct headspace analysis of HCN above *Macadamia* plant parts (flower, leaves, nuts, and husks) was carried out using selected ion flow tube–mass spectrometry (SIFT-MS). The cyanogenic glycoside dhurrin and total cyanide in the extracts were analyzed using HPLC-MS and UV–vis spectrophotometer, respectively. HCN released in the headspace was at a maximum when *Macadamia* samples were treated with pH 7 buffer solution and heated at 50 °C for 60 min. Correspondingly, treatment of *Macadamia* samples under these conditions resulted in 93–100% removal of dhurrin and 81–91% removal of total cyanide in the sample extracts. Hydrolysis of cyanogenic glucosides followed a first-order reaction with respect to HCN production where cyanogenesis is principally induced by pH changes initiating enzymatic hydrolysis rather than thermally induced reactions. The effective processing of different *Macadamia* plant parts is important and beneficial for the safe production and utilization of *Macadamia*-based products.

## 1. Introduction

*Macadamia*-based commercial products have rapidly increased in recent years. In addition to *Macadamia* nuts, *Macadamia* flowers, husks, leaves, and shells are now widely used as a source of functional foods, beverages, and raw materials in cosmetics, feed, and other applications. Abundant antioxidant substances, such as polyphenols, can be extracted from *Macadamia* skin and husks for utilization in the food and pharmaceutical industries [1,2,3]. Bioactive constituents in *Macadamia* are believed to provide health benefits such as improved blood lipid profiles, decreased inflammation, oxidative stress, and reduced cardiovascular disease risk factors [4,5].

**Figure 1:**
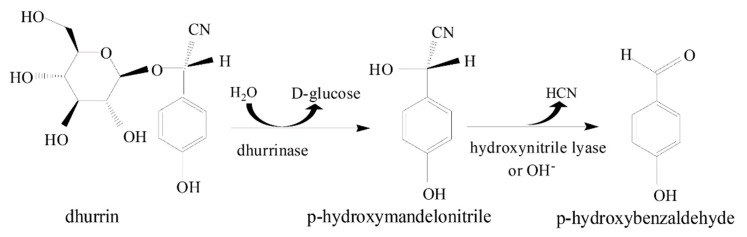
Enzymatic hydrolysis of dhurrin [17,18].

Species within the genus *Macadamia*, however, contain cyanogenic glycoside compounds, which are secondary metabolites that release hydrogen cyanide through cyanogenesis [6,7]. Cyanogenic glycosides are widely distributed in plants and are usually *O*-β-glycosidic derivatives of α-hydroxynitriles originating from the five hydrophobic protein amino acids tyrosine, phenylalanine, valine, leucine, and isoleucine [8,9]. Biological cyanogenesis requires unstable cyanohydrin or a stable cyanogen and degradative enzymes [10,11]. In plants, cyanogenesis mostly occurs via hydrolysis of cyanogenic glycosides that is enzymatically catalyzed by one or more glycosidases and α-hydroxynitrile lyases. These substrates and enzymes are localized in separate vacuoles intact within the subcellular or tissue level of the plant so that under normal physiological conditions, hydrolysis does not occur and autotoxicity is prevented [12,13]. However, tissue disruption allows mixing of substrate and enzymes initiating cyanogenesis and subsequently, hydrolysis [6,7]. Figure 1 shows a two-step enzymatic hydrolysis of dhurrin (4-hydroxymandelonitrile-β-d-glucoside), a cyanogenic glycoside found in *Macadamia.* A more complex biosynthetic pathway for dhurrin hydrolysis is available in the literature [14,15]. In Figure 1, hydrolysis is initially catalyzed by endogenous dhurrinase (a β-D-glucoside glucohydrolase) producing D-glucose and an unstable intermediate, p-hydroxy-(S)-mandelonitrile. The nitrile group is rapidly cleaved off of this intermediate, producing hydrogen cyanide and *p*-hydroxybenzaldehyde, which is catalyzed enzymatically by α-hydroxynitrile lyase [16,17].

Acute and chronic toxicities of hydrogen cyanide from plant-derived food have been reported [16,19,20]. Ingestion of 0.5–3.5 mg cyanide/kg body weight results in acute toxicity. Sublethal doses could lead to headache, hyperventilation, vomiting, weakness, abdominal cramps, and partial circulatory and respiratory systems failure. Moreover, cyanide can inhibit cellular respiration, which could result in fatal poisoning [7,16,21]. The concentration of cyanogenic glycosides, such as dhurrin and proteacin, varies among plant species of *Macadamia* (i.e., *M. ternifolia*, *M. integrifolia*, and *M. tetraphylla*) [6,22]. These compounds are also unevenly distributed within the different parts of a plant (i.e., nuts, seeds, and roots), and their concentrations change at different developmental stages from seed germination to plant maturation [23,24].

In this study, different conditions causing the hydrolysis of cyanogenic compounds in *Macadamia* that subsequently produce hydrogen cyanide gas were evaluated. Most characterization studies in *Macadamia* and other plants only involved analysis of cyanogenic glycosides using time-consuming assays coupled with HPLC analysis [6,25,26]. Furthermore, typical analysis of releasable cyanide uses tedious assays and subsequent spectrophotometry or LC- or GC-MS analysis [14,27,28]. In this study, hydrogen cyanide was measured directly above the headspace of the different parts of the *Macadamia* plant including the flowers, leaves, husks, and nuts using selected ion flow tube–mass spectrometry (SIFT-MS). To our knowledge, this is the first study to measure hydrogen cyanide in real time and directly above the headspace of *Macadamia* samples using SIFT-MS. The rapid and real-time analysis of hydrogen cyanide is particularly important in the processing of the various parts of *Macadamia* that are known to contain cyanogenic glycosides and can subsequently hydrolyze and undergo cyanogenesis. The optimum conditions (heating temperature, heating time, and pH) for the hydrolysis of cyanogenic glycosides via cyanogenesis toward the maximum generation of hydrogen cyanide were determined. Identifying these conditions would be useful in the pre-processing of *Macadamia* to ensure maximum hydrolysis of cyanogenic glucoside, leading to maximum release and volatilization of hydrogen cyanide and ultimately toward the safe production and utilization of *Macadamia*-based products especially as ingredients in food and beverages.

## 2. Materials and Methods

### 2.1. Sample Preparation

*Macadamia* (*M. integrifolia*) flowers, leaves, nuts, and three variety of husks (A16, Oc, and 695) were donated by Shouxiang Township Organic Agricultural Products Development Co., Ltd. (Guangxi, China). The three varieties of *M. integrifolia* husks were introduced and propagated in China from Australia and are hybrid cultivars selected from different plantations or open-pollinated progeny (variety A16). HPLC-grade water, hexane, Na_2_HPO_4_, and citric acid were purchased from Fisher Scientific (Fisher Chemical, Fair Lawn, NJ, USA).

*Macadamia* flowers and leaves were freeze-dried, ground, and sifted. *Macadamia* husks were air-dried, crushed, and sifted. *Macadamia* nuts were crushed, defatted using hexane, and air-dried. All samples were stored in sealed bottles under freezing temperature (−20 °C).

### 2.2. Buffer Preparation

Na_2_HPO_4_ solution (0.2 mol/L) was prepared by dissolving 14.2 g Na_2_HPO_4_ with 500 mL carbon dioxide-free HPLC water. Citric acid solution (0.1 mol/L) was prepared by dissolving 10.51 g citric acid with 500 mL HPLC water.

Different volumes of 0.2 mol/L Na_2_HPO_4_ solution and 0.1 mol/L citric acid were mixed to prepare various buffer solutions with pH 2, 3, 4, 5, 6, 7, 8, and 9. The pH of each solution or sample mixture was measured using a Model 10 pH meter (Denver Instrument Company, Arvada, CO, USA).

### 2.3. Optimization of Heating Temperature and Heating Time

*Macadamia* samples (0.100 g) were subjected to different heating times and temperatures to evaluate the optimum conditions for the maximum hydrolysis of cyanogenic compounds and maximum production of hydrogen cyanide. Samples were heated at 30, 40, 50, 60, 70, or 100 °C. At each temperature, samples were heated for 20, 30, 45, 60, 80, 100, or 120 min. Immediately after heating, the headspace concentration of hydrogen cyanide was measured using SIFT-MS.

### 2.4. Optimization of pH-Buffering Solution

To evaluate the optimum pH for maximum enzymatic activity and the hydrolysis reaction, 0.100 g powdered *Macadamia* flower sample was dissolved in 0.75 mL Na_2_HPO_4_–citric acid buffered solutions with different pH (2, 3, 4, 5, 6, 7, 8, or 9). The solutions were heated at 50 °C for 15, 30, 60, 90, and 120 min. The headspace concentration of hydrogen cyanide was immediately measured using SIFT-MS.

### 2.5. Headspace Cyanide Analysis Using SIFT-MS

Headspace hydrogen cyanide (HCN) was analyzed using a V200 selected ion flow tube–mass spectrometry, SIFT-MS (Syft^TM^ Technologies, Middleton, Christchurch, New Zealand). Using selected ion scan mode, HCN was measured using the H_3_O^+^ precursor ion to detect a protonated HCNH^+^ at *m/z* 28 with a reaction rate, *k*, of 3.8 × 10^−9^ cm^3^s^−1^. SIFT-MS has recently been used for the headspace analysis of various compounds in different food (oil, cheese, and garlic) and breath matrices [29,30,31,32,33,34]. For the headspace detection of HCN using SIFT-MS, 0.100 g of *Macadamia* flowers, leaves, husks, or defatted nuts sample was weighed into individual 500 mL Schott bottles. Then, 0.75 mL HPLC water or Na_2_HPO_4_–citric acid buffer was added, and the solution was mixed and heated (50 °C) in a water bath (Precision Inc., Winchester, VA, USA).

**Figure 2:**
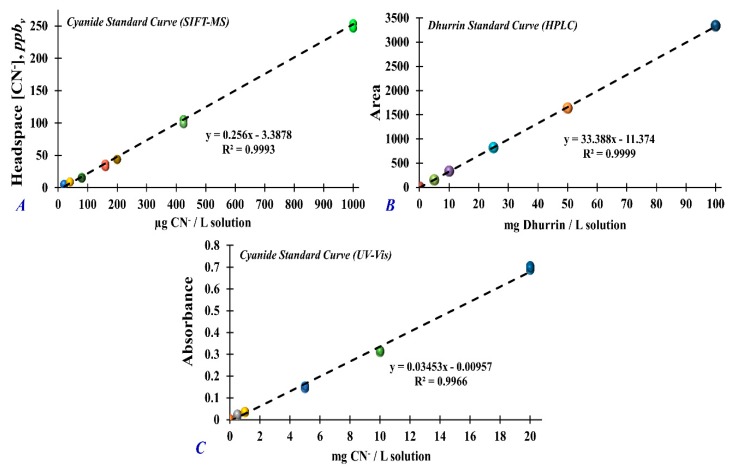
Standard calibration curve and linear regression analysis of (**A**) headspace cyanide concentration (*ppb_v_*) measured by selected ion flow tube–mass spectrometry (SIFT-MS) as a function of cyanide concentration in aqueous solution (µg/L); (**B**) peak area of dhurrin measured by HPLC-DAD (diode array detector) as a function of dhurrin concentration in aqueous solution (mg/L); (**C**) UV–vis spectral absorbance of cyanide standards as a function of cyanide aqueous solution concentration (mg/L). Linear regression analyses were performed at 95% confidence intervals.

A stock cyanide standard solution (1002 ± 5 mg/L KCN in 0.1% NaOH, Specpure, Alfa Aesar, Tewksbury, MA, USA) was used to prepare the working standard aqueous solutions (0, 20, 40, 80, 160, 200, 425, and 1000 µg/L). After this, 1 mL of the working standard solution or matrix blank (HPLC water) was transferred to a 100 mL Schott bottle sealed with a septum-lined screw cap. The working standards were heated at 50 °C for 30 min to allow for headspace equilibrium prior to SIFT-MS analysis. Figure 2A shows the concentration of cyanide in the headspace (*ppb_v_*) as a function of cyanide concentration in aqueous solution (µg/L) generated by a linear regression model. The correlation coefficient (R^2^) for the calibration curve was 0.9993 which signifies that the linear regression model fits the data having <0.0001 significance probability associated with the F statistic (Pr > F) at 95% confidence intervals.

Immediately after achieving headspace equilibrium by heating, headspace sampling was carried out by inserting a passivated sampling needle (~3.5 cm) through the bottle’s septum. The sample inlet flow rate was optimized to 0.35 ± 0.01 Torr·L s^−1^ (26 ± 1 cm^3^ min^−1^ under standard ambient temperature (298 K). The scan duration was 120 s. HPLC water or Na_2_HPO_4_–citric acid buffer was used as a blank solution. Lab air was analyzed in between samples to minimize carry-over effects and potential cross-contamination. Five replicates were performed in all analyses.

### 2.6. Dhurrin Analysis in Plant Extracts Using HPLC

Dhurrin extraction and analysis were performed based on the procedure by De Nicola and co-workers [35]. Briefly, 0.2 g of freeze-dried, powdered plant sample was weighed into a 25 mL centrifuge tube and 0.1 g of activated carbon (Fisher Chemical, Fair Lawn, NJ, USA) and 10 mL methanol (Fisher Chemical, Fair Lawn, NJ, USA) were added. The mixture was sonicated for 25 min at room temperature in a 435 W ultrasonic water bath (Model FS28H, Fisher Scientific, Fair Lawn, NJ, USA) and was left overnight in the tube. After 12–14 h, the mixture was centrifuged (Model Sorvall Legend XFR Centrifuge, Thermo Fisher Scientific, Waltham, MA, USA) for 30 min at 17,000× *g* and 10 °C and was filtered through a Whatman no. 4 filter paper (GE Healthcare, Buckinghamshire, UK). The supernatant was collected and 1:1 (*v/v*) HPLC-grade water was added to the resulting solution. Prior to HPLC analysis, the diluted supernatant solution was filtered through a 0.2 µm RC membrane filter (Phenomenex, Torrance, CA, USA) using a luer-type syringe (Henke-SASS Wolf GmbH, Tuttlingen, Germany) and was transferred into 1.5 mL amber vials for HPLC analysis.

Dhurrin stock standard solution was prepared by dissolving 1 mg of pure dhurrin standard (Sigma Aldrich, St. Louis, MO, USA) with 1 mL of HPLC-grade water. Working standard solutions (0, 5, 10, 25, 50, and 100 mg dhurrin/L solution) were prepared using aliquots of the stock standard solution and diluted with 1:1 H_2_O/methanol (*v/v*) solution. Dhurrin standard solutions were transferred to 1.5 mL amber vials, correspondingly, for HPLC analysis. A solution of 1:1 H_2_O/methanol (*v/v*) was used as matrix blank. Figure 2B shows the peak area of dhurrin as a function of dhurrin concentration in aqueous solution (mg/L) generated by the linear regression model. The correlation coefficient (R^2^) for the calibration curve was 0.9999 which signifies that the linear regression model fits the data having a 0.0037 significance probability associated with the F statistic (Pr > F) at 95% confidence intervals.

**Figure 3:**
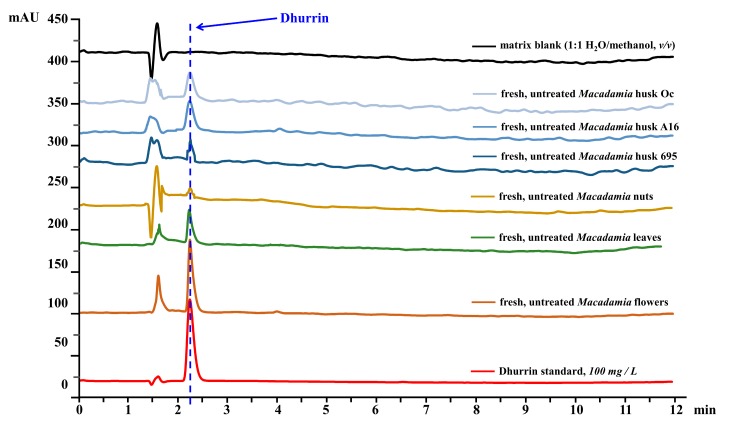
Representative HPLC chromatograms showing the dhurrin spectral peak in 100 mg/L dhurrin standard, matrix blank (1:1 H_2_O/methanol, *v/v*), and extracts of fresh, untreated *Macadamia* flowers, leaves, nuts, husk 695, husk A16, and husk Oc. Dhurrin was monitored at 232 nm absorbance using a diode array detector.

Analysis of dhurrin from the sample extracts and standards were carried out using an HPLC (1100 Series, Agilent Technologies, Santa Clara, CA, USA) equipped with a G1311A quaternary pump, a G1322A degasser, a G1313 ALS autosampler, and a G1316A thermostated column compartment with a C-18 column. The chromatographic conditions involved a flow rate of 1 mL/min by eluting with a gradient of water (A) and acetonitrile (B). The gradient program was set as follows: isocratic 10% B for 1 min, linear gradient to 30% B for 7 min, and linear gradient to 10% B for 2 min. Dhurrin was detected using a G1315B diode array detector (DAD) detector (Agilent Technologies, Santa Clara, CA, USA), and its absorbance was monitored at 232 nm. Dhurrin’s spectral peak was identified by comparing the retention time to that of pure dhurrin from the standard solutions. The resulting chromatograms (Figure 3) were automatically integrated using ChemStation software (Agilent Technologies Inc., Santa Clara, CA, USA). Five replicates per standard or sample extracts were performed in all analyses.

### 2.7. Total Cyanide Analysis in Plant Extracts Using UV–Vis Spectrophotometer

The alkaline picrate method was used for the extraction and analysis of total cyanide as outlined by Sarkiyayi and Agar [36] and Omar and co-workers [37]. Five grams (5 g) dried samples and 50 mL HPLC water were placed in a conical flask which was soaked overnight and then filtered using Whatman no. 4 filter paper. One mL of the filtrate was transferred to a test tube, and 4 mL alkaline picric acid solution was added. The mixture was incubated for 5 min in a 95 °C H_2_O bath. After color development, the absorbance of the mixture was measured at 490 nm using a Varian UV–vis spectrophotometer (Agilent, Cary 50 Bio UV/Visible, Santa Clara, CA, USA). Alkaline picric acid solution was prepared by mixing 1 g picric acid (2,4,6-trinitrophenol crystal, Electron Microscopy Sciences, Hatfield, PA, USA), 5 g Na_2_CO_3_ (Fisher Scientific, Fair Lawn, NJ, USA), and 200 mL HPLC water.

A stock cyanide standard solution (1002 ± 5 mg/L KCN in 0.1% NaOH) was used to prepare the working standard aqueous solutions (0–20 mg/L). One milliliter of the working standard solution or matrix blank (HPLC water) was transferred to a test tube. Four milliliters of alkaline picric acid solution was added, and the mixture was incubated for 5 min in a 95 °C H_2_O bath for color development. The solution absorbance was measured at 490 nm using a UV–vis spectrophotometer. Figure 2C shows the spectral absorbance as a function of the cyanide concentration in aqueous solution (mg/L) generated by the linear regression model. The correlation coefficient (R^2^) for the calibration curve was 0.9966 which signifies that the linear regression model fits the data having <0.0001 significance probability associated with the F statistic (Pr > F) at 95% confidence intervals. For analysis, 20 replicates per standard and 10 replicates per sample extract were used for UV–vis measurement.

### 2.8. Statistical Analysis

Data fitting, analysis of least square means, and regression analysis of the headspace hydrogen cyanide concentrations were carried out using the PROC REG and PROC MIXED options of Statistical Analysis System (SAS^®^ Institute Inc., Cary, NC, USA). Analysis of variance (ANOVA) was performed to analyze the statistical differences of cyanide concentration between different samples using least significant difference of the means (LSD) technique using SAS. Significance was defined using *p* < 0.05 (95% confidence intervals) for least square means comparison. Five replicates were performed in all analyses, except where otherwise specified. Limit of blank (LOB) and limit and detection (LOD) were determined using the methods described by Browne and Whitcomb [38] and Shrivastava and Gupta [39]. The estimated headspace LOB*_(PHBA)_* and LOD*_(PHBA)_* for HCN using SIFT-MS were 2.462 *ppb_v_* and 2.775 *ppb_v_*, respectively, which were determined using repeated headspace measurements of blank (*n* = 60) heated in a water bath at 90 °C for 50 min.

## 3. Results and Discussion

### 3.1. Optimization of Heating Temperature and Time for Maximum Generation of Hydrogen Cyanide

**Figure 4:**
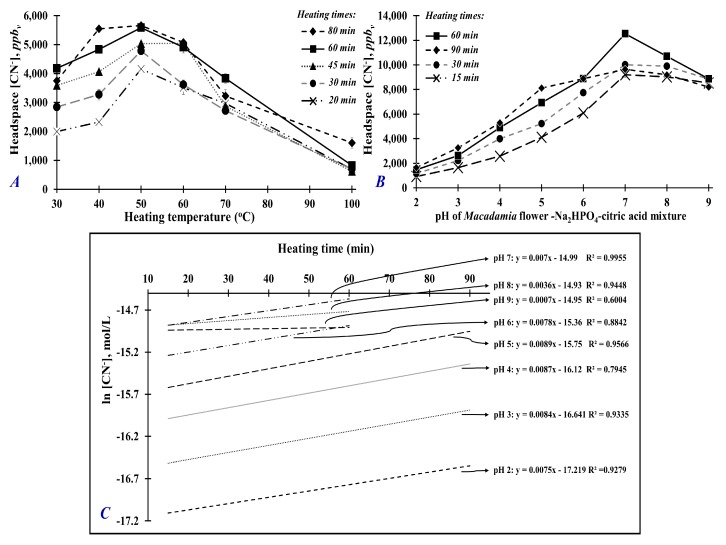
(**A**) Hydrogen cyanide (HCN) concentration in the headspace of *Macadamia* flower heated at various temperatures (30, 40, 50, 60, 70, and 100 °C) and different heating times (20, 30, 45, 60, and 80 min). (**B**) Hydrogen cyanide concentration in the headspace of *Macadamia* flower treated with Na_2_HPO_4_–citric acid mixture buffered at different pH (2, 3, 4, 5, 6, 7, 8, and 9) and heated at 50 °C for 15, 30, 60, and 90 min heating times. (**C**) Hydrolysis reaction rate analysis using [HCN, mol/L] concentration analyzed in the headspace of *Macadamia* flower treated with Na_2_HPO_4_–citric acid mixture buffered at different pH (2, 3, 4, 5, 6, 7, 8, and 9) and heated at 50 °C for 60 min. Standard error analysis was evaluated at 95% confidence intervals.

Figure 4A shows the concentration of hydrogen cyanide (HCN) from the headspace of *Macadamia* flower samples heated at 30, 40, 50, 60, 70, and 100 °C for 20, 30, 45, 60, and 80 min. For all heating times, the headspace HCN concentration increased from 30 to 50 °C and decreased linearly beyond 50 °C. Thus, HCN generation in *Macadamia* was maximum at 50 °C. These results suggest that the optimum temperature for enzymatic activity of endogenous dhurrinase and α-hydroxynitrile lyase is 50 °C (Figure 1). At higher heating temperatures (i.e., above 50 °C), cyanide production decreased, which could be caused by decreased enzyme activity or inactivation and, therefore, reduced subsequent hydrolysis reaction of the main cyanogenic glycoside dhurrin (Figure 4A). It is interesting to note that the decreasing production of cyanide at higher temperatures (60–100 °C) is gradual rather than an abrupt cyanide reduction that could be expected from thermally induced enzyme inactivation. The measured cyanide at high temperature could be produced from other thermolabile cyanogenic glycosides that are present in minor amounts. At high temperature, the isomers of dhurrin such as taxiphyllin, zierin, and *p*-glucosyloxy-mandelonitrile can readily dissociate and release cyanide without enzymatic hydrolysis [40,41,42]. Therefore, the cyanide released from the thermally induced decomposition of these minor cyanogenic glycosides could be contributing to the detected cyanide in the headspace of *Macadamia* flower samples heated at higher temperatures.

In addition, the longer the heating time, the higher the HCN concentration, with the longest heating times (60 and 80 min) generating the highest HCN concentration (Figure 4A). The maximum HCN concentration was reached when samples were heated at 40–50 °C for 80 min or 50 °C for 60 min. From these results, the optimum heating time and temperature were determined to be 50 °C for 60 min, which were used for succeeding experiments.

### 3.2. Optimization of Mixture’s pH for Maximum Generation of Hydrogen Cyanide

Figure 4B shows the headspace concentration of hydrogen cyanide above *Macadamia* flower samples treated with Na_2_HPO_4_–citric acid buffered solutions at different pH (2, 3, 4, 5, 6, 7, 8, 9) and heated at 50 °C for 15, 30, 60, and 90 min. As the pH increased from pH 2 to 7, the concentration of HCN increased. From pH 7 to 9, the concentration of HCN decreased slightly. The *Macadamia* flower sample treated with pH 7 buffer and heated at 50 °C for 60 min generated the highest headspace concentration of HCN. This result suggests that these conditions are optimum for the underlying enzymatic activities involved in the hydrolysis reaction of cyanogenic glycoside compounds producing hydrogen cyanide gas (Figure 1).

When *Macadamia* flower was heated at 50 °C for 60 min under its normal physiological pH (pH 4.35), the HCN level was only about 4900–5600 *ppb_v_* (Figure 4A,B). Increasing the treatment’s pH to pH 7, significantly increased the headspace HCN concentration by 200–250% (~12,500 *ppb_v_*). At a more basic pH (pH 8 or 9), HCN concentration was still significantly higher than the concentration at acidic pH (pH 6 and below), but it was lower than that at pH 7.

Further analysis of data was done by plotting the hydrogen cyanide concentration as a function of pH (Figure 4C), the hydrolysis reaction of cyanogenic glycosides at 50 °C could be described as a first-order reaction with respect to the production of hydrogen cyanide. A constant pseudo first-order rate value (*k* = 0.0081 ± 0.0007 M min^−1^) was determined from the linear regression slopes of ln [HCN], mol L^−1^ versus heating time (min) plots for pH 2, 3, 4, 5, 6, and 7. At pH 8 and 9, the 90 min data point had to be excluded. The calculated empirical rates of hydrogen cyanide production (d[HCN]/d*t*) at different pH (Table 1) suggest that hydrogen cyanide production is slower at acidic pH values (pH 2, 3, 4, 5, and 6), increases at basic pH, but reaches a peak at pH 7. These findings are similar to the results of the study by Johansen and co-workers [17]. According to their study, hydrolysis of the cyanogenic glycoside, dhurrin, follows a first-order reaction with respect to dhurrin and the rate of dhurrin hydrolysis is very slow at low pH values but strongly increases as the pH is increased. Thus, the first-order rate of hydrolysis of cyanogenic glycoside dhurrin in aqueous solution is supported by the in vitro hydrolysis of cyanogenic glycosides in *Macadamia* flower as reported by the present study.

### 3.3. Headspace Concentration of Hydrogen Cyanide above *Macadamia* Flowers, Leaves, Nuts, and Husks

**Figure 5:**
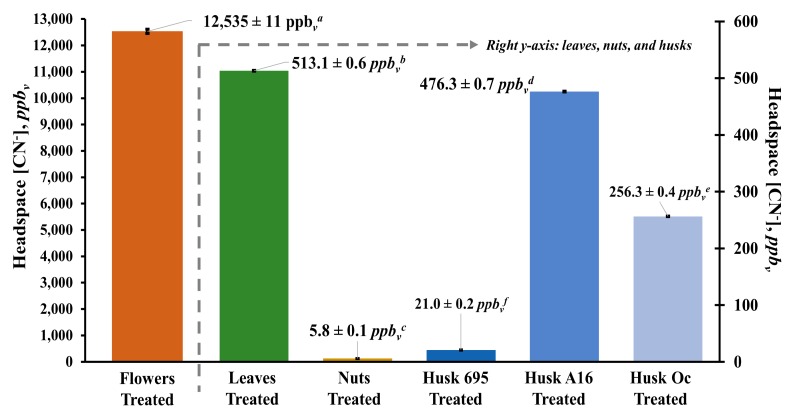
Hydrogen cyanide (HCN) concentration directly analyzed in the headspace of *Macadamia* flowers, leaves, nuts, and three varieties of *Macadamia* husks (695, A16, and Oc) treated with Na_2_HPO_4_–citric acid mixture buffered at pH 7 and heated at 50 °C for 60 min. Different superscript letters (*a*, *b*, *c*, *d*, *e*, or *f*) indicate significant differences in mean HCN concentration at *p* < 0.05 (95% confidence intervals).

Figure 5 shows the headspace concentration of hydrogen cyanide measured by SIFT-MS directly above the *Macadamia* samples treated with pH 7 buffered solution and heated at 50 °C for 60 min. Based on this figure, *Macadamia* flowers produced the significantly highest levels of hydrogen cyanide (12,535 ± 11 *ppb_v_*), followed by the leaves (513 ± 0.6 *ppb_v_*), husks (21 ± 0.1 *ppb_v_*, (695); 256 ± 0.4 *ppb_v_*, (Oc); 476 ± 0.7 *ppb_v_*, (A16), and nuts (5.8 ± 0.1 *ppb_v_*) at *p* < 0.05. Interestingly, the hydrogen cyanide concentration in *Macadamia* flowers was approximately 2–3 times higher than the highest concentration of cyanide detected in *M. ternifolia* cotyledon (~4800–6500 *ppb_v_*) [6,43]. Previous reports have mentioned that cyanogenic compounds are highest in growing tissues of plants and that activation of metabolic processes coincide with cyanogenic glycoside production [6,44]. The flower is the main reproductive organ of a plant and has very active and complex morphological and physiological features, which support an abundance of ecological functions related to floral development and plant reproduction [45,46,47]. For instance, de novo synthesis of amino acids, enzymes, and structural proteins, which are precursors of N-containing secondary metabolites (such as cyanogenic glycosides) and signaling molecules, all occur in floral tissues [47]. These complex metabolic processes during floral development and growth could be contributing to the increased biosynthesis of cyanogenic glycosides resulting in the higher concentration of hydrogen cyanide generated in the flower than in the other parts of *Macadamia*.

The concentration of hydrogen cyanide in *Macadamia* leaves (513 ± 0.6 *ppb_v_*) is within the concentration range of cyanide (364–1403 *ppb_v_*) detected in the leaf tissue of *M. ternifolia, M. integrifolia,* and *M. tetraphylla* species during their early to mid-developmental stages (3rd–4th week) [6]. Young leaves were observed to contain higher amounts of cyanogenic glycosides, which could be due to the copious amounts of carbon and nitrogen precursors readily available during germination, so there is rapid biosynthesis of cyanogenic compounds. Cyanogenic glycoside in leaf tissue was, however, observed to decrease with plant maturation because these compounds are rapidly metabolized and broken down as the leaves become older [6,48,49,50].

*Macadamia* husks are the fleshy green fibrous pericarp covering the conical or spherical hard brown shell enclosure of *Macadamia* nuts [51]. Similar to *Macadamia* flowers, there are no available published data reported for the hydrogen cyanide concentration in *Macadamia* husks for comparison. Moreover, the hydrogen cyanide concentration of husks analyzed from three different *Macadamia* varieties were significantly different: 21 ± 0.1 *ppb_v_* for variety 695; 256 ± 0.4 *ppb_v_* for variety Oc; and 476 ± 0.7 *ppb_v_* for variety A16 (Figure 5). It was previously reported that the quantities of cyanogenic glycosides in *Macadamia* seedlings and other plants vary according to species, developmental stage, and tissue type; however, the cyanogenic glycosides in the varieties of *Macadamia* husk used in this study have yet to be conclusively identified [6,52].

Seeds of *Macadamia* species are also capable of accumulating cyanogenic glycoside compounds and the concentration varies depending on the variety [6,43]. In the present study, the hydrogen cyanide concentration of *Macadamia* nuts (5.8 ± 0.1 *ppb_v_*) was lower than the reported cyanide concentrations in commercially used seeds (~74 *ppb_v_*) of *M. integrifolia* or *M. tetraphylla* and significantly lower than the concentration detected in *M. ternifolia* (~4800 *ppb_v_*), which is considered to be inedible.

### 3.4. Dhurrin and Total Cyanide Concentrations in Untreated and Treated *Macadamia* Plant Part Extracts

**Figure 6:**
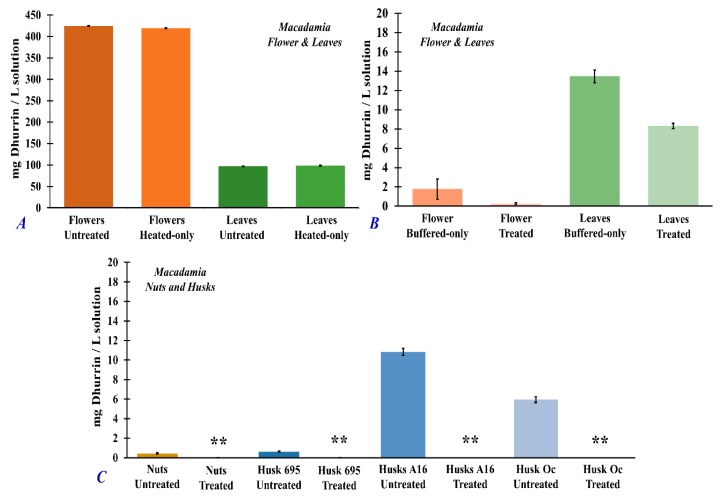
Dhurrin concentration (mg dhurrin/L solution) measured by HPLC-DAD from the different extracts of *Macadamia* plant parts ((**A**,**B**) flowers and leaves; (**C**) nuts and *husks varieties 695,* A16, and Oc) in untreated samples (fresh, no treatment), heated-only samples (50 °C for 60 min), buffered-only samples (samples treated with pH 7, Na_2_HPO_4_–citric acid buffer solution), and treated samples (samples treated with pH 7, Na_2_HPO_4_–citric acid buffer solution and heated at 50 °C for 60 min). ** Means negligible values. Standard error analysis was evaluated at 95% confidence intervals.

Figure 6 shows the concentration of cyanogenic glycoside dhurrin from the different *Macadamia* plant part extracts with different treatments. Fresh, untreated *Macadamia* flower (424.4 ± 0.6 mg/L) had the highest amount of dhurrin compared to the fresh, untreated leaves (97.1 ± 0.6 mg/L) (Figure 6A), fresh nuts (0.43 ± 0.07 mg/L), or the different husk varieties (695: 0.63 ± 0.04 mg/L; A16: 11.1 ± 0.3 mg/L; Oc: 6.1 ± 0.3 mg/L) (Figure 6C).

**Figure 7:**
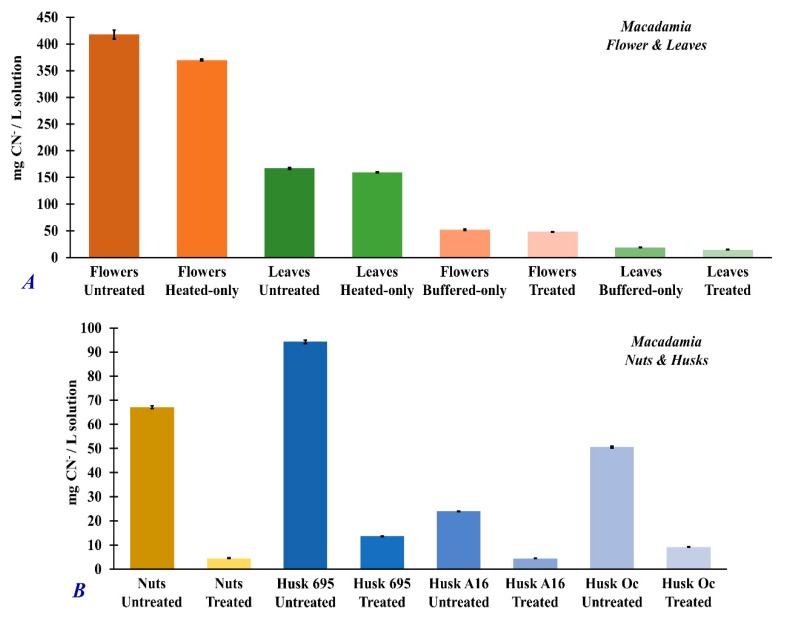
Total cyanide concentration (mg CN^−^/L solution) measured by UV–vis spectrophotometer from the different extracts of *Macadamia* plant parts ((**A**) flowers and leaves; (**B**) nuts and *husk varieties 695, A16, and Oc*) in untreated samples (fresh, no treatment), heated-only samples (samples heated at 50 °C for 60 min), buffered-only samples (samples treated with pH 7, Na_2_HPO_4_–citric acid buffer solution), and treated samples (samples treated with pH 7, Na_2_HPO_4_–citric acid buffer solution, and heated at 50 °C for 60 min). Standard error analysis was evaluated at 95% confidence intervals.

The cyanide concentration in the extracts have the same trend as the dhurrin concentration. Figure 7 shows the total cyanide concentration of the fresh, untreated *Macadamia* flower (417.7± 0.8 mg/L), leaves (167 ± 2 mg/L), nuts (67.1 ± 0.6 mg/L), and husks (695: 94.3 ± 0.7 mg/L; A16: 23.9 ± 0.1 mg/L; Oc: 50.6 ± 0.4 mg/L). After full sample treatment using the optimized conditions (i.e., samples treated with pH 7 buffer solution and heated at 50 °C for 60 min), significant amounts of dhurrin and cyanide were removed in the analyzed extracts as shown in Figure 6 and Figure 7, respectively.

It is interesting to note that heating samples at 50 °C for 60 min without pH adjustment (heated-only samples) had little to no effect on the removal of dhurrin (Figure 6A) or cyanide (Figure 7A) in *Macadamia* flower and leaf samples. On the other hand, treating the *Macadamia* flower and leaf samples with buffered solution at pH 7 without heating (buffered-only), resulted in significant removal of dhurrin (Figure 6B: flower, 419 ± 1 mg/L; leaves, 98 ± 1 mg/L) and cyanide (Figure 7A: flower, 370 ± 2 mg/L; leaves, 48 ± 1 mg/L) in the extracts. Analysis of treatment efficiencies (Table 2) showed that the full treatment of samples (i.e., treated samples) by heating (50 °C, 60 min) and pH 7 adjustment results in 93–100% removal of dhurrin and about 81–91% removal of cyanide in the different *Macadamia* plant parts. Treatment by heating alone was only about 1% effective in the removal of dhurrin and only about 5–12% effective in the removal of cyanide (Table 2). However, treating the *Macadamia* flower and leaf samples with buffered solution at pH 7 without heating (buffered-only), treatment produced about similar removal effectivity (Table 2) of dhurrin (93–100%) and cyanide (89–91%) as that of the fully treated samples heated at 50 °C for 60 min at pH 7.

## 4. Conclusions

The optimum conditions for the maximum release of hydrogen cyanide in *Macadamia* samples were 50 °C, 60 min at pH 7. Under these treatment conditions, trace amounts of hydrogen cyanide could still be detected in the headspace directly above the different *Macadamia* plant part samples using SIFT-MS. The measured hydrogen cyanide in the headspace of the treated samples were 12,535 ± 11 *ppb_v_* (flower), 513 ± 0.6 *ppb_v_* (leaves), 6 ± 0.1 *ppb_v_* (nuts), 476 ± 0.7 *ppb_v_* (husk A16), 256 ± 0.4 *ppb_v_* (husk Oc), and 21 ± 0.1 *ppb_v_* (husk 695). Treatment of *Macadamia* samples under these optimum conditions produced 93–100% removal of dhurrin and 81–91% removal of total cyanide in the sample extracts. Treatment by pH 7 adjustment (buffered-only) without heating also resulted in an effective removal of dhurrin (86–100%) and total cyanide (88–89%) in *Macadamia* extract similar to the full, optimized treatment conditions. Heating the samples alone at 50 °C for 60 min without pH adjustment was not effective in the hydrolysis and removal of cyanogenic glycoside dhurrin and total cyanide in *Macadamia* samples. The varying concentration of generated hydrogen cyanide could be correspondingly attributed to the concentration of cyanogenic glycosides (such as dhurrin) from the different parts of the *Macadamia* plant and their subsequent hydrolysis to hydrogen cyanide. Cyanogenic glycosides were greatest in *Macadamia* flowers, followed by the leaves and husks (depending on variety), and lowest in nuts. The results indicate that the hydrolysis of cyanogenic glycosides in *Macadamia* is predominantly induced by pH changes rather than by heat. This further suggests that the enzymatic hydrolysis involved in cyanogenesis is chiefly pH-directed rather than thermally induced. In addition, the hydrolysis reaction of cyanogenic glycosides could be described as a first-order reaction with respect to the in vitro production of hydrogen cyanide.

These results provide further insights into the cyanogenic systems in *Macadamia*. Moreover, the evaluated optimum conditions for the hydrolysis of dhurrin and removal and release of hydrogen cyanide could be helpful for the effective processing of different parts of *Macadamia*. Such information provides some guidelines toward the safe production, utilization, and consumption of *Macadamia*-based products.

## Figures and Tables

**Table 1:** Empirical rates of [HCN] production of *Macadamia flower* treated with Na_2_HPO_4_–citric acid mixture buffered at different pH and heated at 50 °C for 60 min.

pH of *Macadamia* Flower–Na_2_HPO_4_–Citric Acid Buffered Mixture	Empirical Rate of [HCN] Production at 50 °C (d[HCN]/d*t*), M·L^−1^·min^−1^
2	4.61 × 10^−10^ ± 0.2 × 10^−10^
3	8.25 × 10^−10^ ± 0.2 × 10^−10^
4	1.95 × 10^−09^ ± 0.07 × 10^−09^
5	2.36 × 10^−09^ ± 0.08 × 10^−09^
6	2.34 × 10^−09^ ± 0.08 × 10^−09^
7	2.79 × 10^−10^ ± 2 × 10^−10^
8	1.40 × 10^−09^ ± 0.2 × 10^−09^
9	2.78 × 10^−10^ ± 4 × 10^−10^

**Table 2:** Dhurrin and total cyanide (mg/g sample) in untreated and treated *Macadamia* flowers, leaves, nuts, and husk varieties and treatment removal efficiency (%).

Samples	mg Dhurrin/g Sample	% Treatment Efficiency	Total Cyanide (mg/g sample)	% Treatment Efficiency
*Macadamia* Flower Untreated	37.14 (±0.05)	---	4.18 (±8.0 × 10^−2^)	---
Flowers (heated only)	36.68 (±0.09)	1.2 (±0.5)	3.70 (±7.5 × 10^−3^)	11.5 (±0.2)
Flowers (buffered only)	0.15 (±0.09)	99.6 (±0.5)	0.52 (±1.1 × 10^−2^)	87.6 (±2.9)
Flowers Treated	0.02 (±0.09)	99.9 (±0.5)	0.48 (±3.6 × 10^−4^)	88.5 (±2.9)
*Macadamia* Leaves Untreated	8.50 (±0.05)	---	1.67 (±1.8 × 10^−2^)	---
Leaves (heated only)	8.60 (±0.09)	1.2 (±0.5)	1.59 (±7.5 × 10^−3^)	4.6 (±0.2)
Leaves (buffered only)	1.18 (±0.06)	86.1 (±0.3)	0.19 (±3.3 × 10^−3^)	88.7 (±1.6)
Leaves Treated	0.73 (±0.09)	93.1 (±0.5)	0.15 (±1.6 × 10^−3^)	91.3 (±1.6)
*Macadamia* Nuts Untreated	0.04 (±0.07)	---	0.67 (±6.3 × 10^−3^)	---
Nuts Treated	0.00 (±0.09)	100.0 (±0.5)	0.05 (±2.2 × 10^−3^)	93.2 (±1.4)
*Macadamia* Husks 695 Untreated	0.05 (±0.07)	---	0.94 (±7.4 × 10^−3^)	---
Husks 695 Treated	0.00 (±0.09)	100.0 (±0.5)	0.14 (±2.9 × 10^−4^)	85.5 (±1.2)
*Macadamia* Husks A16 Untreated	0.97 (±0.07)	---	0.24 (±1.2 × 10^−3^)	---
Husks A16 Treated	0.00 (±0.09)	100.0 (±0.5)	0.04 (±3.6 × 10^−4^)	81.3 (±0.9)
*Macadamia* Husks Oc Untreated	0.53 (±0.07)	---	0.51 (±4.1 × 10^−3^)	---
Husks Oc Treated	0.00 (±0.09)	100.0 (±0.5)	0.09 (±3.1 × 10^−4^)	81.7 (±1.3)

* Standard error analysis was evaluated at 95% confidence intervals.
